# Uncertainty quantification of the impact of peripheral arterial disease on abdominal aortic aneurysms in blood flow simulations

**DOI:** 10.1098/rsif.2023.0656

**Published:** 2024-04-10

**Authors:** Sharp C. Y. Lo, Jon W. S. McCullough, Xiao Xue, Peter V. Coveney

**Affiliations:** ^1^ Centre for Computational Science, University College London, London, UK; ^2^ Advanced Research Computing Centre, University College London, London, UK; ^3^ Informatics Institute, Faculty of Science, University of Amsterdam, Amsterdam, The Netherlands

**Keywords:** abdominal aortic aneurysm, peripheral arterial disease, wall shear stress, blood flow simulation, uncertainty quantification, sensitivity analysis

## Abstract

Peripheral arterial disease (PAD) and abdominal aortic aneurysms (AAAs) often coexist and pose significant risks of mortality, yet their mutual interactions remain largely unexplored. Here, we introduce a fluid mechanics model designed to simulate the haemodynamic impact of PAD on AAA-associated risk factors. Our focus lies on quantifying the uncertainty inherent in controlling the flow rates within PAD-affected vessels and predicting AAA risk factors derived from wall shear stress. We perform a sensitivity analysis on nine critical model parameters through simulations of three-dimensional blood flow within a comprehensive arterial geometry. Our results show effective control of the flow rates using two-element Windkessel models, although specific outlets need attention. Quantities of interest like endothelial cell activation potential (ECAP) and relative residence time are instructive for identifying high-risk regions, with ECAP showing greater reliability and adaptability. Our analysis reveals that the uncertainty in the quantities of interest is 187% of that of the input parameters. Notably, parameters governing the amplitude and frequency of the inlet velocity exert the strongest influence on the risk factors’ variability and warrant precise determination. This study forms the foundation for patient-specific simulations involving PAD and AAAs which should ultimately improve patient outcomes and reduce associated mortality rates.

## 1. Introduction

An abdominal aortic aneurysm (AAA) is a permanent and localized dilation of the abdominal aorta [1]. Human-centred surveys have revealed a range of 3 to 8% for the prevalence of AAAs in the global population across different settings [2–4]. Although AAAs are usually asymptomatic [1,3], the growth of AAAs can increase the risk of rupture [1,5] which leads to an overall mortality rate of 86% [6]. Therefore, the medical profession needs to improve its understanding of the risk factors for the growth and rupture of AAAs.

It is known that wall shear stress (WSS) plays an important role in both the growth and rupture of AAAs. Endothelial cells of the aortic wall are highly responsive to WSS [7]. Mechanical stimuli on these cells may increase the activity of proteolytic enzymes and disrupt the balance between the synthesis and degradation of the tissue constituents, resulting in the growth of an aneurysm [1,7,8]. If such an imbalance is highly localized, the aneurysm may even rupture [9]. Indeed, previous studies have shown that the growth and rupture of aneurysms are associated with regions of abnormal WSS [9–12].

Many patients with an AAA also suffer from stenosis, occlusion, or vessel dilation in a daughter branch of the aorta [13–15], which is referred to as peripheral arterial disease (PAD) [4]. It is widely accepted that PAD is a risk factor for the growth and rupture of AAAs [2,16,17]. While there are many population-based screening studies of the correlation between PAD and AAAs [16], the interactions between PAD and AAAs have rarely been studied directly. It is of special interest to know whether the changes in blood flow due to PAD have significant effects on the growth and rupture of AAAs in the general population.

Computational fluid dynamics (CFD) is useful for studying these effects as it allows non-invasive analysis of blood flow in various settings [18]. The lattice Boltzmann method (LBM) [19,20] is attractive for the simulation of blood flow since it is applicable to complex geometries and highly scalable on supercomputers [21–23]. Previous works have used LBM in modelling the effects of the wall motion [24,25], blood rheology [26,27] and pulsatile blood flow [26,28]. In addition, LBM has proved effective for simulating blood flow in arteries with aneurysms [29].

However, to accurately describe such complex fluid flow generally requires a model with many degrees of freedom. While the model parameters can be determined based on experimental and clinical data, obtaining such data is often unfeasible or costly. Therefore, it is necessary to identify which parameters can be fixed in all simulations (parameter fixing) and which parameters are the most important to determine precisely (parameter prioritization) [30]. These can be achieved by performing a sensitivity analysis in the framework of uncertainty quantification to determine the relative importance of the model parameters with respect to the quantities of interest (QoIs) [31–33].

In this paper, we present a fluid mechanics model designed for simulating the effects of PAD on the risk factors associated with the growth and rupture of AAAs. The effects of PAD on blood flow are modelled by specifying abnormal flow rates in the peripheral arteries as the boundary conditions of the blood flow simulations, whereas the risk factors are assessed by some QoIs related to WSS. Through analysis of the peripheral flow rates and AAA risk factors in CFD simulations performed within a comprehensive arterial geometry featuring an AAA, this study aims to (i) quantify the uncertainty in controlling the flow rates in peripheral vessels and investigate its sensitivity to model parameters, (ii) quantify the uncertainty in predicting the AAA risk factors and investigate its sensitivity to model parameters, and (iii) identify the model parameters that can be fixed and those that require more precise determination. The results of this study will enhance the understanding of the uncertainty inherent in the proposed fluid mechanics model, facilitate its validation, and contribute to investigating the haemodynamic interactions between PAD and AAAs.

## 2. Methods

Let M be a deterministic simulation model which admits a vector of input parameters X and generates some QoIs Y=M(X). If the space X is uncertain, the uncertainty will propagate to Y. The goal of forward uncertainty quantification is to obtain informative metrics related to the probability distribution of Y arising from this propagation of uncertainty. By studying the variations of Y in response to those of X, we can understand the relative importance of each input in X to each quantity of interest in Y. This procedure is known as sensitivity analysis.

In this section, we describe the setup of the sensitivity analysis of our fluid mechanics model for simulating the effects of PAD on the risk factors for the growth and rupture of AAAs. We start with the simulation domain in §2.1 and the simulation model in §2.2. Then we describe the QoIs in §2.3 and the input parameters in §2.4. After that, we present the approach for uncertainty propagation in §2.5 and the approach for sensitivity analysis in §2.6. Lastly, we introduce a metric used to assess the amplification of the model uncertainty in §2.7.

### 2.1. Simulation domain

**Figure 1.**
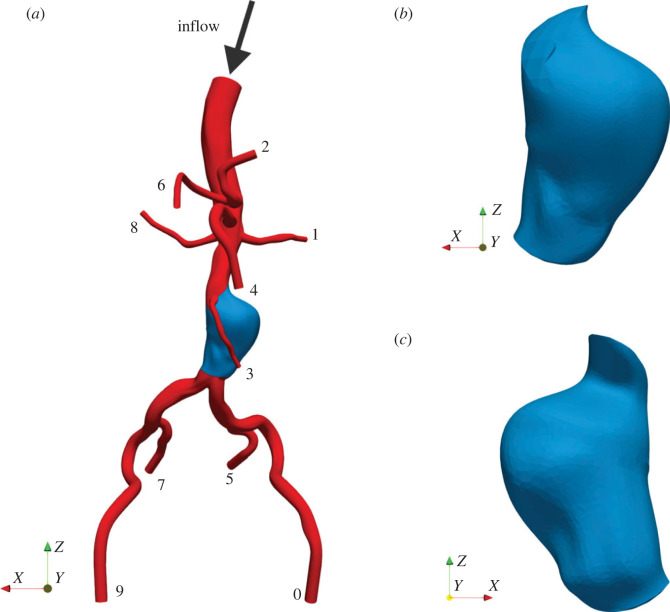
(*a*) The full model of the abdominal aorta with an abdominal aortic aneurysm (AAA), (*b*) the front side of the AAA and (*c*) the back side of the AAA. The AAA is coloured in blue, and the indices of the outlets are labelled.

We use the arterial model shown in figure 1 as the domain for our simulations of blood flow. It extends from the suprarenal aorta to the iliac arteries, covering the main peripheral branches around the AAA. The arteries with outlet indices running from 0 to 9 are the left external iliac, left renal, splenic, inferior mesenteric, superior mesenteric, left internal iliac, hepatic, right internal iliac, right renal and right external iliac arteries, respectively. The AAA is located at the infrarenal aorta.

This arterial model is obtained by modifying the 0156_0001 model in the Vascular Model Repository [34]. The original model was produced from a computed tomography angiogram of a 69-year-old male human. Since its units are not specified in the manual, we assume it is in millimetres and scale it up six times in each dimension to meet the general size of the human artery. The sharp turns near outlets 0, 4, 7 and 9 are trimmed to avoid the risk of instability in the simulations. Subsequently, all openings are elongated such that the flow direction is approximately normal to the boundary planes to fulfil the assumptions of the boundary conditions. We note that manual operations on the artery model are sometimes inevitable due to the imperfection in the segmentation of medical images [35]. Indeed, the above modifications are found to substantially improve the stability and mass conservation of our simulations.

The arterial model consists of one inlet with an equivalent radius of 7.27 mm and 10 outlets with radii ranging from 1.12 mm at Outlet 3 to 2.91 mm at Outlet 0. The smallest vessel, Outlet 3, has a length of 36.7 mm. The longest diameter of the AAA is 26.6 mm.

In the construction of the simulation domain, the arterial model is voxelized [36] to create a three-dimensional grid of uniformly spaced lattice sites representing the fluid region. A resolution of Δ*x* = 100 μm is chosen so that there are about 11 lattice sites along the equivalent radius of the smallest vessel. As a result, there are 44.3 million lattice sites in the entire simulation domain. To ensure this resolution is high enough to produce reliable results, we compare the risk factors observed on the AAA walls in the simulations using this grid and those using a finer grid (Δ*x* = 70.7 μm; see electronic supplementary material). While there are small differences in the magnitudes observed, there are no significant differences in their spatial distributions. Since our analysis focuses on identifying the high-risk regions with extreme values, the similarities in the distribution of risk factors mean that we are justified in using the coarser domain for our campaigns. The differences in the observed magnitudes of these extremes have little impact on the conclusions drawn.

### 2.2. Simulation model

We perform the simulations of three-dimensional blood flow using HemeLB [37,38], an open-source fluid flow solver based on the LBM. The code has been verified through order-of-accuracy tests [39] and was validated in previous works examining vascular flows [27,40]. The LBM is established on kinetic theory which describes the statistical behaviour of particles at the mesoscopic scale [19,20]. While conventional methods directly solve for the macroscopic variables in the Navier–Stokes equations, the LBM solves for the velocity distribution function *f* of fluid particles in the Boltzmann equation. The discretization of *f* in the phase space of time *t*, position x and particle velocity introduces a number of *q* populations $\left\{f_{i},i=0,\ldots ,q\right\}$ with discrete velocities $\left\{c_{i}\right\}$. Subsequently, the evolution of {*f*_*i*_} is governed by the lattice Boltzmann equations

2.1 $$f_{i}(t+\Delta t,x+c_{i}\Delta t)=f_{i}(t,x)+\Omega_{i}(t,x)\;\Delta t,$$

where Ω is the collision operator, which characterizes the interactions occurring between particles. These equations can be shown to replicate the incompressible Navier–Stokes equations in regimes with a sufficiently low Mach number $Ma=\|U\|/c_{s}$, where $c_{s}=(1/\sqrt{3})\Delta x/\Delta t$ is the speed of sound. The modelling errors associated with this process, or compressibility errors, typically scale with $O(Ma^{2})$. Macroscopic fields such as the fluid density *ρ*, flow velocity U and fluid pressure *P* can be calculated using the populations

2.2 $$\rho =\sum_{i}f_{i},$$

2.3 $$U=\frac{1}{\rho }\sum_{i}f_{i}c_{i},$$

2.4 $$P=\rho c_{s}^{2}.$$

In this paper, we use a three-dimensional lattice with 19 velocities, designated D3Q19, and the two-relaxation-time (TRT) collision operator as they are a compromise between accuracy and efficiency [20]. In the TRT framework, the symmetric relaxation time *τ*^+^ is linked to the dynamic viscosity of the fluid *μ*, whereas the anti-symmetric relaxation time *τ*^−^ is used to fix the so-called ‘magic’ parameter Λ, which characterizes the truncation error and stability properties of the TRT model [41,42]. For the D3Q19 lattice, the relation between *τ*^+^ and *μ* is

2.5 $$\mu =\rho c_{s}^{2}(\tau^{+}-\frac{\Delta t}{2}).$$

The magic parameter has the form

2.6 $$\Lambda =(\frac{\tau^{+}}{\Delta t}-\frac{1}{2})(\frac{\tau^{-}}{\Delta t}-\frac{1}{2}).$$

We discuss the choice of these parameters in §2.4.

The motion of vessel walls has a noticeable impact on blood flow. To fully capture the behaviour of blood flow in elastic vessels requires a coupled model of fluid–structure interactions in general, yet performing such a simulation can be computationally costly. Alternatively, we use the wall boundary condition for LBM developed by McCullough & Coveney [25] which captures the key features of elastic-walled Womersley flows more accurately than a rigid wall implementation. This approach also preserves the inherent locality of LBM, rendering it computationally efficient. The idea behind this implementation is to mimic the effect of an expanding wall on the flow in the bulk by enforcing a slip velocity at the wall that is representative of the velocity at that location if a no-slip wall had moved beyond that point. The expansion of the vessel of radius *r* is related to the fluid pressure *P* by

2.7 $$\Delta r=\frac{(1-\sigma^{2})r^{2}}{Eh}(P-P_{0}),$$

where *σ*, *E*, and *h* are Poisson’s ratio, Young’s modulus, and thickness of the vessel wall, respectively, and *P*_0_ is the fluid pressure when Δ*r* = 0. The unknown incoming distribution functions at fluid sites next to the wall are calculated using the extrapolated velocity at extended wall locations. This calculation involves the parameter boundary velocity ratio *F*, which is defined as the ratio of the velocity at the extended wall location to the velocity at the fluid site (see electronic supplementary material and [25,43] for more detail). We explain the choice of these parameters in §2.4.

We impose a time-dependent velocity boundary condition on the inlet using the same approach in our previous work [44]. The flow velocity at the centre is set to follow a profile composed of a warm-up period and 10 periods of a real heartbeat profile in the descending aorta found in the literature [45]. The peak velocity $U_{\max}$ and the angular frequency *ω*_0_ of the heartbeat profile are calculated from the Reynolds number (Re) and the Womersley number (Wo), respectively, using the relations

2.8 $$Re=\frac{\rho U_{\max}r}{\mu }$$

and

**Figure 2.**
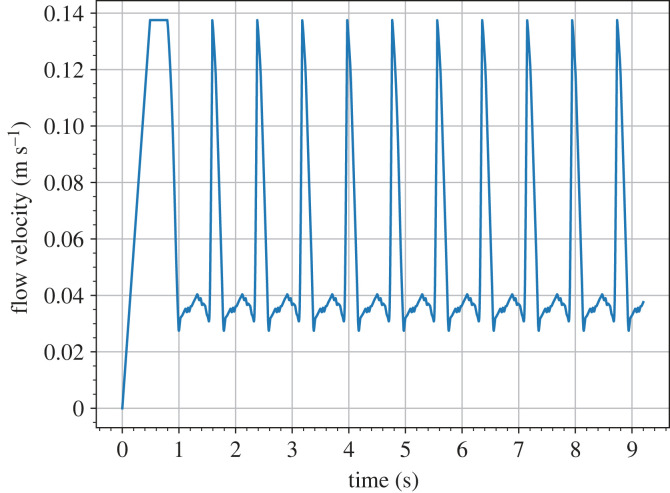
Mean profile of the flow velocity at the centre of the inlet. It is composed by one warm-up period and 10 heartbeats. In this setting, Re=600 and Wo=11.2.

2.9 $$Wo=r\sqrt{\frac{\rho \omega_{0}}{\mu }},$$

where *r* is the equivalent radius of the inlet. The values of Re and Wo are sampled from some distributions presented in §2.4 for studying their impacts on the risk factors for the AAA. Figure 2 shows the velocity profile substituted with the means of the distributions, Re=600 and Wo=11.2. From a more general perspective, HemeLB has the capability to custom-generate the spatial profile imposed at an inlet by specifying a weighting at each lattice site, which is then applied to the temporal velocity profile.

Regarding the boundary conditions for the outlets, we impose ratios of the flow rates (*Q*) using the strategy proposed in our previous work [44]. An advantage of this strategy is that the number of free parameters stays constant as the number of outlets increases, leading to a low cost of parameter calibration. In this strategy, each outlet is coupled with the two-element Windkessel (WK2) model which calculates the fluid pressure *P* from the flow rate *Q* by solving the differential equation

2.10 $$P+RC\frac{dP}{dt}=RQ.$$

The resistance *R* and capacitance *C* in these WK2 models are determined from the desired *Q* ratios and the fundamental frequency of the pulsation, *ω*_0_, as well as the free parameters *γ*_*R*_ and *γ*_*C*_. Previous findings [44,46] suggest that the *Q* ratios can attain the desired values if the scaling factor for the resistance *γ*_*R*_ is larger than a certain threshold. In addition, the stability and convergence rate of the simulations can be controlled by the scaling factor for the capacitance *γ*_*C*_. We discuss the choice of the desired *Q* ratios and the values of *γ*_*R*_ and *γ*_*C*_ in §2.4.

### 2.3. Quantities of interest

There are two kinds of QoIs in this study: the measured flow rate ratios at the outlets and the risk factors for the growth and rupture of AAAs.

#### 2.3.1. Measured flow rate ratios

**Figure 3.**
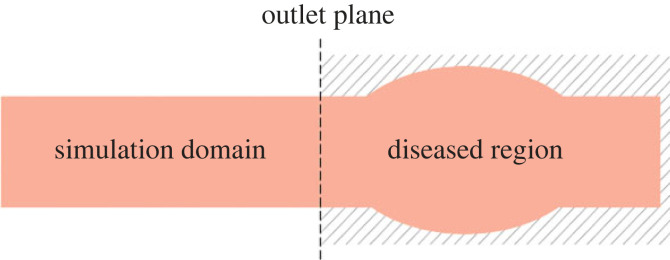
Schematic diagram of an outlet vessel with PAD. The diseased region is in the same vessel as the outlet but outside the simulation domain.

PAD refers to stenotic, occlusive and aneurysmal diseases of the aorta and its non-coronary branches [4]. These diseases manifest as the contraction or expansion of peripheral vessels which leads to a decrease or increase in flow rates, *Q*. Due to mass conservation, the flow rate does not change across the region of contraction or expansion. Hence, this region can be thought to lie outside the boundary of the domain when a boundary condition for the flow rate is used (figure 3). Therefore, the effects of PAD on blood flow can be modelled by specifying abnormal values of the flow rates at the outlets of undistorted peripheral vessels.

As detailed in §2.2, we use the strategy developed in a previous study [44] to impose the desired *Q* ratios on the outlets. Although this approach is shown to be effective in some simulations of blood flow [44,46], its validation is an ongoing process which requires many test cases representing different scenarios. In this regard, we perform simulations of blood flow in the abdominal aorta of a patient with an AAA in the rest condition. Therefore, the measured *Q* ratios at the outlets in the simulations are QoIs and are compared with the desired *Q* ratios in the analysis.

The measured flow rate in an outlet is obtained during the post-processing of simulations. It is obtained by integrating the normal component of the flow velocity, U, over the boundary plane:

2.11 $$Q=\int U\cdot \hat{n}\;dA,$$

where $\hat{n}$ and *A* are the unit normal vector and area of the boundary plane, respectively. The same approach is used in the WK2 models in our simulations.

In the calculations of the measured *Q* ratios, the flow rates are normalized with respect to the reference outlet, Outlet 2 (see §2.4.4), before averaging. Their relative errors compared with the desired *Q* ratios are also calculated, where the base effect of the desired *Q* ratios and the effect of the reference outlet are eliminated.

#### 2.3.2. Risk factors for abdominal aortic aneurysms

Both high and low WSS can disrupt the imbalance between the synthesis and degradation of the extracellular matrix in the aortic wall, thereby promoting the growth and rupture of aneurysms; however, as suggested by Meng *et al*. [9], high and low WSS induce the imbalance through different pathways. In the case of an aneurysm experiencing an impinging flow, high WSS and a positive WSS gradient can trigger an imbalance involving the mural cells. On the other hand, in the presence of a circulatory flow, low and oscillatory WSS can lead to an imbalance involving the inflammatory cells.

As can be seen in figure 1*a*, the orientation of the AAA compared to that of the entrance and exit of the main aortic vessel suggests that the preferential flow path is not impinging directly on the walls of the AAA. The diverging nature of the flow to fill the AAA is more conducive to circulatory flow developing within this region [12]. We confirm this by plotting the velocity vectors observed at the walls of the AAA (see electronic supplementary material). Therefore, we focus on the second pathway and the associated WSS quantities.

The magnitude of WSS is commonly measured using time-averaged wall shear stress (TAWSS). TAWSS is computed by averaging the norm of the WSS vector $\tau_{w}$ (see electronic supplementary material and [47,48] for more detail) over the duration of the measurement. Suppose the measurement starts at time *T*_*s*_ and finishes at time *T*_*f*_. Then,

2.12 $$\text{TAWSS}=\frac{1}{T_{f}-T_{s}}\int_{T_{s}}^{T_{f}}\|\tau_{w}\|\;dt.$$

In a study involving 295 patients with AAAs, Bappoo *et al.* [11] showed that major adverse aneurysm-related events are more likely to occur in patients with a TAWSS of 0.4 Pa or less. Moreover, in the measurements from 18 male patients with AAAs using four-dimensional magnetic resonance images of the aorta, Trenti *et al.* [49] found that the mean TAWSS in the infrarenal aorta was 0.15 ± 0.02 Pa. Similar results were observed in some patient-specific CFD simulations [50–52]. Therefore, low WSS can be defined as the mean value minus two standard deviations, i.e. 0.11 Pa.

The oscillation of WSS is often assessed using the oscillatory shear index (OSI) [53], given by

2.13 $$\text{OSI}=\frac{1}{2}(1-\frac{\|\int_{T_{s}}^{T_{f}}\tau_{w}\;dt\|}{\int_{T_{s}}^{T_{f}}\|\tau_{w}\|\;dt}).$$

OSI is a dimensionless quantity ranging from 0, when the WSS vector is collinear with the TAWSS vector throughout the measurement, to 0.5, when the TAWSS vector vanishes. A mean value of around 0.20 ± 0.05 in an AAA was observed in the measurements by Trenti *et al.* [49] and in some patient-specific CFD simulations [50–52]. Hence, OSI can be considered high if it exceeds 0.30.

To measure the effects of both the magnitude and oscillation of WSS, Di Achille *et al.* [54] proposed using the ratio of TAWSS and OSI, called the endothelial cell activation potential (ECAP):

2.14 $$\text{ECAP}=\frac{\text{OSI}}{\text{TAWSS}}.$$

By construction, a lower WSS or a higher OSI results in a higher ECAP. Thus, a higher ECAP indicates a higher risk of the growth and rupture of AAAs. With the above thresholds of TAWSS and OSI, a high ECAP can be defined as one with a value exceeding 2.7 Pa^−1^.

A prolonged interaction between circulating inflammatory cells and the endothelium can facilitate the degradation of the wall [9]. This happens if the fluid particles reside close to the aortic wall for an extended period. To measure the duration, Himburg *et al.* [55] proposed a metric called the relative residence time (RRT), given by

2.15 $$\text{RRT}=\|\frac{1}{T_{f}-T_{s}}\int_{T_{s}}^{T_{f}}\tau_{w}\;dt{\|}^{-1}=[(1-2\cdot \text{OSI})\cdot \text{TAWSS}{]}^{-1}.$$

By substituting the above threshold values of TAWSS and OSI into this equation, we obtain an upper limit of 22.7 Pa^−1^ above which RRT is regarded as high.

### 2.4. Input parameters

Here, we discuss the choice of the input parameters for the simulation model presented in §2.2. There are a total of 17 parameters, nine of which are varied. We present a summary of the choice in table 1 and explain it in the following sections.

**Table 1.** Input parameters of the simulation model. Note that the period of a heartbeat *T*_0_ is a function of the Womersley number Wo.

parameter	range of values	distribution	unit	involved in
Δ*x*	100	constant	μm	voxelization
Δ*t*	60	constant	μs	TRT model
Λ	[0.0036, 0.25]	log-uniform	dimensionless	TRT model
*ρ*	1050	constant	kg m^−3^	fluid property
*μ*	0.0035	constant	Pa s	fluid property
Re	[540, 660]	uniform	dimensionless	inlet boundary condition
Wo	[10.1, 12.3]	uniform	dimensionless	inlet boundary condition
*σ*	0.5	constant	dimensionless	elastic wall model
$\hat{r}$	2.015	constant	mm	elastic wall model
*h*	1.775	constant	mm	elastic wall model
*E*	[3.32, 27.57]	uniform	MPa	elastic wall model
*F*	[0.32, 0.76]	uniform	dimensionless	elastic wall model
*m*	[1, 4]	uniform	dimensionless	outlet boundary condition
*γ*_*R*_	[2^8^, 2^12^]	log-uniform	dimensionless	outlet boundary condition
*γ*_*C*_	[2^−4^, 2^0^]	log-uniform	dimensionless	outlet boundary condition
*t*_*f*_	9.2	constant	s	simulation time
*T*_*s*_	[*t*_*f*_ − 4*T*_0_, *t*_*f*_ − 3*T*_0_]	uniform	s	measurements

#### 2.4.1. Parameters for fluid flow

The density of the blood, *ρ*, is assumed to be 1050 kg m^−3^ [56]. The dynamic viscosity of the blood, *μ*, is assumed to have a constant value of 0.0035 Pa s, which is a valid assumption in the aorta [57].

The Reynolds number Re and the Womersley number Wo are used to characterize the shape of the velocity profile at the inlet of the abdominal aorta (figure 1*a*). The typical values of Re and Wo in the abdominal aorta of a person at rest are 600 and 11.2 respectively [58,59]. These values correspond to a peak velocity of 0.139 m s^−1^ and a period of 0.774 s of the velocity profile, which is shown in figure 2.

As we have no preference for the values in the space, we assume the values of Re and Wo follow a uniform distribution centred at 600 and 11.2, respectively. The upper and lower limits are defined as 90% and 110% of the mean values.

#### 2.4.2. Parameters for two-relaxation-time model

Given the values of Δ*x*, *μ* and *ρ*, there are two degrees of freedom concerning the TRT collision operator. The first is related to *τ*^+^, and its value is determined under the criterion for the compressibility error of LBM [19,20]. Since the compressibility error scales with $Ma^{2}$ which depends on ‖U‖ and Δ*t*, a suitable value of Δ*t* must be chosen such that $Ma^{2}$ is sufficiently small for all flow speeds encountered. Using the maximum flow speed at the inlet as a guide, we set Δ*t* to 60 μs, or equivalently *τ*^+^/Δ*t* = 0.56 based on equation (2.5). We track the level of $Ma^{2}$ throughout the simulations and report it in §3.

The second degree of freedom is related to *τ*^−^, but its value is determined from Λ via equation (2.6). It is known that Λ has effects on both the accuracy and the stability of the simulation [20]. Prior studies have found some special values which are optimal in different situations. For example, Λ=1/12 cancels the third-order truncation error; Λ=1/6 cancels the fourth-order truncation error; Λ=3/16 results in the middle position between walls and fluid nodes for the bounce-back scheme. In particular, Λ=1/4 provides the optimal stability in the sense that the stability bound of the flow is determined by solely the flow velocity but not the relaxation parameters [42]. Moreover, this optimal stability subclass was extended to a wider range of Λ with the limits 1/8 and 1/4 [60]. Besides, the TRT model reduces to the single-relaxation-time model, also known as the BGK model [61], when *τ*^+^ = *τ*^−^. By substituting $\tau^{+}=\tau^{-}=0.56\Delta t$ into equation (2.6), we obtain Λ=0.0036.

Since the interval [0.0036, 0.25] covers all the above special values, we define it to be the sample space of Λ. To effectively draw samples in this large range, we assume Λ follows a log-uniform distribution in this interval; this means that $\log_{a}\Lambda$ with any base *a* is uniformly distributed.

#### 2.4.3. Parameters for elastic wall model

The mechanical properties of the vessels are assumed to be uniform throughout the arterial model. Therefore, equation (2.7) is applied to all lattice sites near the walls with the same parameter values and *r* is replaced with the effective vessel radius $\hat{r}$.

We set Poisson’s ratio, *σ*, to be 0.5 since biological tissues are practically incompressible [62]. We determine the value of $\hat{r}$ using the size of the inlet and outlets. The lower and upper quartiles are 1.37 mm (Outlet 8) and 2.66 mm (Outlet 9), respectively. Therefore, we assume $\hat{r}=2.015\;\mathrm{mm}$ with 32% uncertainty. According to the measurements by Rosero *et al.* [63], the age-adjusted median aortic wall thickness of men is 1.58–1.97 mm within a 95% confidence interval; this implies a mean value of *h* = 1.775 mm with 11% uncertainty. Van’t Veer *et al.* [64] found that Young’s modulus of the AAA in the studied male patients is 9.0 ± 2.5 MPa.

Since *r*, *h*, *E* are multiplied together in equation (2.7), we hold $\hat{r}$ and *h* constant while varying *E* with all their uncertainties considered. This allows us to reduce the dimension of the sampling space. The above data suggest that E∈[3.32,27.57] MPa with fixed values of $\hat{r}$ and *h*. Likewise, we sample the values of *E* from a uniform distribution defined in this interval.

Boundary velocity value, *F*, is dependent on the Womersley number, Wo, of the flow [25]. To determine suitable values of *F*, we plot its value against percentage wall extension for different values of Wo in electronic supplementary material, figure S1. In physiological conditions, the diameter of the aorta varies by approximately 6% in a cardiac cycle [65,66]. Therefore, we select the values of *F* in the interval [0.32, 0.76] which corresponds to an extension of 2–10% when Wo is 10.1–12.3. Similarly, we sample the values of *F* from a uniform distribution defined in this interval.

#### 2.4.4. Desired flow rate ratios

While it is possible to assume an individual distribution of the desired *Q* at the outlets, a large number of simulations are required to sample the whole parameter space. In this preliminary investigation of the parameter space, we use the following approach to arrive at a manageable number of simulations.

Based on the principle of energy minimization, Murray [67] proposed that the flow rate should be proportional to the third power of the radius in any section of a vessel, i.e. *Q* ∝ *r*^3^. Although this does not hold true in general, studies have established the power relation *Q* ∝ *r*^*m*^ with different values of *m* for different vessels [68,69]. This implies the proportional relationship between the flow rate *Q*_*j*_ in an arbitrary Outlet *j* with an equivalent radius *r*_*j*_ and that in the reference outlet:

2.16 $$\frac{Q_{j}}{Q_{\mathrm{ref}}}=\frac{r_{j}^{m}}{r_{\mathrm{ref}}^{m}}.$$

To minimize the range of the ratios, we choose Outlet 2 which has the median radius among all outlets to be the reference outlet. Using this equation to calculate the desired *Q* ratios at the outlets, we can vary *Q* in all outlets by changing one single value of *m*.

Considering the limited availability of data specifically pertaining to the abdominal aorta, and taking into account that studies have consistently reported values of *m* falling within the range of 1 to 4 for various arteries [68,69], we sample the values of *m* from a uniform distribution spanning the interval of [1, 4].

#### 2.4.5. Scaling factors for two-element Windkessel parameters

The results of our previous work [44] showed that the differences between the measured *Q* ratios and the desired values decreased exponentially when *γ*_*R*_ increased above a certain threshold. In the simulations using the *profunda femoris* model, the differences were smaller than 7% when *γ*_*R*_ = 2^10^. Therefore, in this study, we sample the values of *γ*_*R*_ between 2^8^ and 2^12^ in a log-uniform distribution.

The previous findings also showed that simulations are stable when *γ*_*C*_ is larger than a threshold value [44]. In the current study, we performed a few preliminary tests and found that the simulations were stable for 2^−4^ ≤ *γ*_*C*_ ≤ 2^0^. Therefore, we sample its value from a log-uniform distribution defined in this interval.

#### 2.4.6. Measurements in simulations

Since the blood flow in our simulations is driven by the inlet velocity, we seek a periodic solution to the flow. In preliminary tests, the unsteady blood flow attains a quasi-stationary state after about four heartbeats. Therefore, we set the final time *t*_*f*_ of all simulations to be the end of the 10th heartbeat in the mean profile, which is 9.2 s (figure 2). The flow variables in the last three cycles of each simulation are used to calculate the QoIs. Thus, the duration of measurement *T*_*f*_ − *T*_*s*_ = 3*T*_0_, where *T*_0_ = 2*π*/*ω*_0_ is the period of the heartbeat.

One might question the aleatoric uncertainty in the blood flow since turbulent flow patterns can be found at an intermediate Reynolds number in complex geometry [70]. The chaotic nature of turbulence is often regarded as intrinsic randomness; therefore, the fluctuations caused by turbulence are categorized as aleatoric uncertainty [71].

These fluctuations can be divided into two parts. The first part is related to the fluctuations that occur at the same phase of the periodic solution. As these fluctuations have similar characteristics in general, they can be reduced statistically by averaging the flow quantities over periods of time. The aforementioned time-averaging is expected to reduce this uncertainty in all QoIs. The second part is related to the transience of fluctuations. It is possible that earlier fluctuations have impacts on the present flow dynamics. Put another way, fluctuations that occur at different phases may be correlated to a certain extent. Therefore, the choice of the starting phase of the time averaging may affect the time-averaged results and contain uncertainty.

To examine the impact of this uncertainty on the QoIs, we vary the starting time of the measurements *T*_*s*_ while keeping the length of the measurements constant. Specifically, the measurements start between the last three and four periods of the heartbeats and continue for three periods. Therefore, *T*_*s*_ is varied uniformly between *t*_*f*_ − 4*T*_0_ and *t*_*f*_ − 3*T*_0_.

### 2.5. Uncertainty propagation by polynomial chaos expansion

In uncertainty quantification, uncertainty propagation is a fundamental step performed to characterize the effects of uncertain input parameters (X) on the output QoIs (Y). In the context of three-dimensional blood flow simulations, one of the significant outcomes of interest is the spatial distribution of quantities on the aortic walls. To simplify the analysis, we focus on statistical moments rather than the full probability distribution of these quantities.

A commonly employed approach for this purpose is the method of polynomial chaos expansion (PCE) [31,72,73]. A key advantage of PCE is its non-intrusive nature, as it does not require any specific modifications to the numerical code. Compared to Monte Carlo methods that estimate the full probability distribution, PCE is computationally more efficient while still providing comparable results in moment-based analyses [30,31,33]. However, PCE suffers from the curse of dimensionality: the number of samples becomes large when the number of uncertain parameters is O(10) [30]. We arrive at a manageable number of samples as detailed below with the nine input variables.

The basic concept of the PCE method involves approximating the outputs using a set of basis polynomial functions. To simplify notation, let us assume the output is a scalar value *Y*, although it can be a vector of quantities in general. This output is represented by a series expansion as follows:

2.17 $$Y\approx M^{\mathrm{PCE}}(X)=\sum_{\alpha }w_{\alpha }\Psi_{\alpha }(X),$$

where $w_{\alpha }$ and $\Psi_{\alpha }(X)$ are the expansion coefficient and the basis polynomial function of degree α∈N, respectively. Since the input parameters are assumed to follow a uniform distribution (see §2.4), the Legendre polynomials are used to compose $\left\{\Psi_{\alpha }\right\}$ to optimize the convergence rate of PCE [72].

The coefficients $\left\{w_{\alpha }\right\}$ are determined by the samples of {X,Y} obtained from the model evaluations. We solve for these coefficients by using the regression method because it requires a manageable number of model evaluations and gives more accurate results than the alternative projection method [33]. Suppose polynomial functions up to degree *p* are used and there are *n* input parameters, i.e. $X=\{X_{1},\ldots ,X_{n}\}$. Equation (2.17) can be written as

2.18 $$Y=\sum_{\alpha =0}^{N_{p}-1}w_{\alpha }\Psi_{\alpha }(X)+\varepsilon_{p},$$

where $\varepsilon_{p}$ is the truncation error and

2.19 $$N_{p}=\left(\begin{array}{c} n+p\\ \;p \end{array}\right)$$

is the number of unknown expansion coefficients. The following minimization problem is solved to obtain the solution of $\left\{w_{\alpha }\right\}$:

2.20 $$\{\hat{w_{\alpha }}\}=\arg\min_{\left\{w_{\alpha }\right\}}\;E[\varepsilon_{p}^{2}]=\arg\min_{\left\{w_{\alpha }\right\}}\;E[(M-\sum_{\alpha =0}^{N_{p}-1}w_{\alpha }\Psi_{\alpha }{)}^{2}].$$

The statistical moments of the underlying distribution of *Y* can be calculated by using the coefficients. The equations for the mean and variance are, respectively,

2.21 $$E[Y]=\hat{w_{0}}$$

and

2.22 $$V[Y]=\sum_{\alpha =1}^{N_{p}-1}{\hat{w_{\alpha }}}^{2}.$$

According to Sudret [31], a PCE of degree *p* = 2 is usually sufficient for obtaining accurate estimates of these moments. Therefore, we conduct two campaigns of uncertainty quantification, one using *p* = 2 and the other using *p* = 3, to assess the convergence of the results. In solving the minimization problem in equation (2.20), 2*N*_*p*_ samples are drawn; this is the recommended minimum size for obtaining an accurate solution [30]. With *n* = 9 input variables, 110 and 440 samples are drawn for the campaign with *p* = 2 and *p* = 3, respectively, according to equation (2.19).

### 2.6. Sensitivity analysis by Sobol’ indices

To quantify the relative importance of the input parameters to the QoIs, we perform a sensitivity analysis by using the Sobol’ indices. Sobol’ indices are global, variance-based sensitivity indices that can be used to quantify the contribution of individual input parameters and their interactions to the variance of the output quantities [30,31]. For PCE, the Sobol’ indices can be calculated at a low cost by using the PCE coefficients obtained in uncertainty propagation [30,74]. Below, we describe the main Sobol’ indices used in this study, assuming the output is a scalar value *Y*.

The first-order Sobol’ index *S*_*i*_ measures the direct effect of *X*_*i*_ on the variance of *Y* and is defined as

2.23 $$S_{i}=\frac{V[E[Y|X_{i}]]}{V[Y]},$$

where $E[Y|X_{i}]$ denotes the conditional expected value of *Y* given a fixed value of *X*_*i*_. The second-order Sobol’ index *S*_*ij*_ measures the interactive effect of *X*_*i*_ and *X*_*j*_ on the variance of *Y* and is defined as

2.24 $$S_{ij}=\frac{V[E[Y|X_{i},X_{j}]]}{V[Y]},$$

where $E[Y|X_{i},X_{j}]$ denotes the conditional expected value of *Y* given some fixed values of *X*_*i*_ and *X*_*j*_. Higher-order Sobol’ indices are defined analogously to measure the interactive effects of multiple inputs on the variance of *Y*. As these indices quantify the effects of the variability of the input parameters on the variance of the output, they are useful for deciding which parameters should be determined more precisely (parameter prioritization).

Moreover, the total Sobol’ index *S*_*T*,*i*_ measures both the direct and interactive effects of *X*_*i*_ on the variance of *Y*. It is defined as

2.25 $$S_{T,i}=1-\frac{V[E[Y|X_{∼i}]]}{V[Y]},$$

where *X*_∼*i*_ is the set of all input parameters except *X*_*i*_. A small value of *S*_*T*,*i*_ means the uncertainty in *X*_*i*_ has little effect on the uncertainty in *Y* and hence *X*_*i*_ can be fixed. Therefore, this index is useful for deciding which parameters can be fixed (parameter fixing).

In this work, we use EasyVVUQ and QCG-PilotJob to conduct the campaigns of uncertainty quantification. EasyVVUQ [75,76] is a Python package designed to streamline verification, validation, and uncertainty quantification for simulations. It handles sampling, model evaluation, result aggregation, and analysis in a campaign. Complementing this, QCG-PilotJob [77] facilitates dynamic task execution in a single allocation on computing clusters. Developed as part of the VECMA toolkit [78] and being advanced within the SEAVEA project (https://www.seavea-project.org/), these tools enable efficient and reliable computational job handling. We employ the implementations of PCE and Sobol’ sensitivity analysis in EasyVVUQ.

### 2.7. Coefficient of variation

The coefficient of variation (CV) is a measure of the variability of a probability distribution. A larger CV implies a larger extent to which the distribution is stretched in relation to the mean, and vice versa. The CV of a random variable *X* is defined as the ratio of the standard deviation to the mean of its underlying distribution. The equation is

2.26 $$\text{CV}(X)=\sqrt{\frac{V[X]}{E{\left[X\right]}^{2}}}.$$

Since the CV is dimensionless, it can be compared between different measurements.

An important part of the uncertainty quantification of a model is assessing the amplification of the uncertainty in the outputs with respect to the uncertainty in the inputs. For this assessment, we adopt the coefficient of variation ratio (CVR) proposed by Edeling *et al.* [79]. The CVR of a quantity of interest, *Y*, is defined as the ratio of the CV of *Y* to the average CV of the input variables, X. The equation is

2.27 $$\text{CVR}(Y)=\text{CV}(Y)/\frac{1}{n}\sum_{i}^{n}\text{CV}(X_{i})=\sqrt{\frac{V[Y]}{E{\left[Y\right]}^{2}}}/\frac{1}{n}\sum_{i}^{n}\sqrt{\frac{V[X_{i}]}{E{\left[X_{i}\right]}^{2}}}.$$

A CVR smaller than one indicates diminution of uncertainty, whereas a CVR greater than one indicates amplification.

## 3. Results and discussion

The campaigns of uncertainty quantification were performed on the ARCHER2 UK national supercomputer. The workflow for each sample finished within 40 minutes using 4096 CPU cores on 64 nodes on average. In all simulations of blood flow, the maximum Mach number in all space and time is below 0.2. Hence, the compressibility errors of the simulated flows, which scale with $Ma^{2}=0.04$, are sufficiently small. All simulations reach a quasi-stationary state in the last four heartbeats within which measurements are taken.

In the following subsections, we discuss the results of the uncertainty quantification of the flow rate ratios in outlets and the risk factors for the AAA. The displayed results are obtained from the third-order PCE. They have negligible differences compared with the results obtained from the second-order PCE, and the differences do not affect our conclusions. All the results of our analyses are provided in the electronic supplementary material. It is important to highlight that these collective results represent the entirety of the input parameter space.

### 3.1. Flow rate ratios in outlets

**Figure 4.**
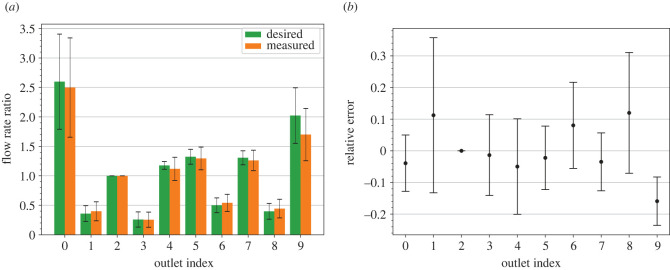
Comparison between the desired and measured *Q* ratios at the respective outlets. (*a*) The targeted and measured *Q* ratios. (*b*) The relative error in the measured *Q* ratios. Each column centres the ensemble mean within a range of 2 s.d. The error for Outlet 2 is consistently zero as it serves as the reference outlet.

The desired and measured *Q* ratios at the outlets are compared in figure 4. As shown in figure 4*a*, the measured *Q* ratios are quite close to the desired ratios at all outlets and their variations are similar. The largest variations occur at Outlets 0 and 9, which correspond to the external iliac arteries (figure 1). Since the desired *Q* ratios are determined by Murray’s power law (equation (2.16)), they have larger variations at the outlets with larger radii. Figure 4*b* shows that the means of the relative error are within 0.17 at all outlets and the standard deviations are about 0.11 in general. In particular, Outlet 9 has the largest relative error, and the interval defined by its mean ± 1 s.d. does not include zero. Hence, it is more difficult to control the flow rate in Outlet 9 accurately. Similarly, Outlet 1 has the largest standard deviation of the relative error; therefore, it is more difficult to control the flow rate in Outlet 1 precisely.

**Figure 5.**
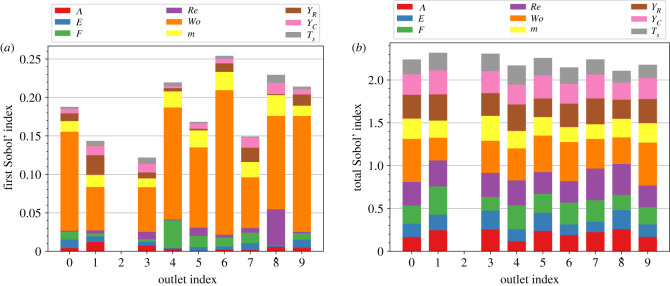
(*a*) First-order and (*b*) total Sobol’ indices of the relative error in the measured *Q* ratios at the respective outlets. Outlet 2 is excluded from the indices as it serves as the reference outlet. The first-order indices highlight the need for a more accurate determination of the Womersley number, Wo. However, the total indices do not pinpoint which input parameter can be held constant.

The first-order and the total Sobol’ indices of the relative error in the measured *Q* ratios are plotted in figure 5*a*,*b,* respectively. The Womersley number, Wo, gives the highest first-order Sobol’ index at all outlets. This result suggests that the Womersley number is the major factor for the variations in the measured *Q* ratios and should be determined more precisely. In addition, the Sobol’ index for the starting time of the measurements, *T*_*s*_, is relatively small at all outlets, suggesting that the simulated flow reaches a nearly stationary state. However, every input parameter gives a similar total Sobol’ index at all outlets. Therefore, there is no preference as to which input parameter should be fixed.

The fact that the Womersley number contributes the most to the uncertainty in the measured *Q* ratios may be related to the simplification that the desired *Q* ratios are time-independent. The Womersley number governs the frequency of the pulsation at the inlet. Since the distance between the inlet and each outlet is different, the pulsation in each outlet has different phases in general. Therefore, when Wo is varied, the *Q* ratios between outlets will change. This variability can be reduced by using time-dependent values for the resistance of the WK2 models [46].

### 3.2. Risk factors for the abdominal aortic aneurysm

Next, we present the results of the risk factors for the growth and rupture of the AAA. We discuss different aspects of the risk factors: spatial distribution on the walls of the AAA, CV and sensitivity to the input parameters.

#### 3.2.1. Spatial distribution on aortic walls

**Figure 6.**
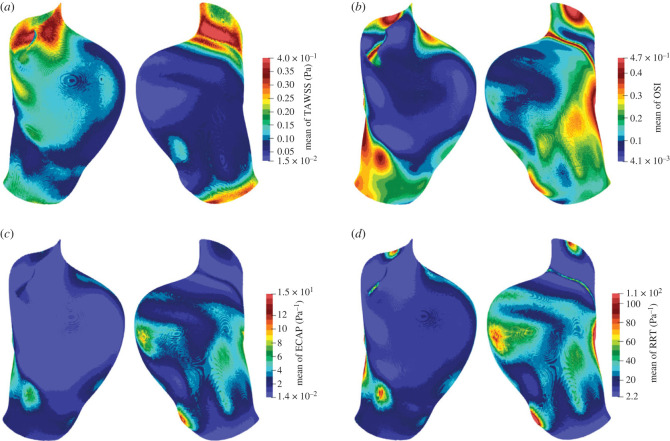
Distribution of the ensemble mean of the risk factors (*a*) TAWSS, (*b*) OSI, (*c*) ECAP and (*d*) RRT on the walls of the abdominal aortic aneurysm. In each panel, the front side of the aneurysm is displayed on the left, and the back side is displayed on the right. The risk factors are averaged over three cardiac cycles in the simulations. There are two regions on the back side where ECAP and RRT exceed the threshold values.

Figure 6 shows the ensemble mean of TAWSS, OSI, ECAP and RRT at each lattice site on the walls of the AAA. TAWSS is lower in the bulge area, where the flow speed is lower due to the larger cross-sectional area, and higher near the entrance and the exit, where the flow speed is higher due to the smaller cross-sectional area. In most of the bulge area, TAWSS is lower than the threshold value of 0.11 Pa. We find a smaller OSI in the bulge area and larger in the surrounding region. However, it is high (above 0.30) on the flat surface opposite the bulge area. ECAP is generally low on the front side. Values higher than the threshold value of 2.7 Pa^−1^ are found mostly at the back: there are two focal regions where ECAP is above 7 Pa^−1^. The distribution of RRT is very similar to that of ECAP. However, the focal regions of high values (above 22.7 Pa^−1^) are more prominent.

The above findings suggest that it is important to consider both the magnitude and degree of oscillation of WSS in assessing the risk of an aneurysm. Either TAWSS or OSI considers only one factor and may give a broad range of regions at risk. On the contrary, ECAP and RRT consider both factors and make the regions at risk stand out.

The similarity of the distributions of ECAP and RRT can be explained by the fact that ECAP and RRT have similar equations (see equations (2.14) and (2.15)). However, one important difference is that the distribution of RRT has a much larger spread. RRT has the factor (1 − 2 · OSI)^−1^ which has the codomain [1, ∞), whereas ECAP is simply proportional to OSI which is between 0 and 0.5. The large spread may pose practical challenges in analyses such as selecting the proper range of values to study.

#### 3.2.2. Coefficient of variation

**Figure 7.**
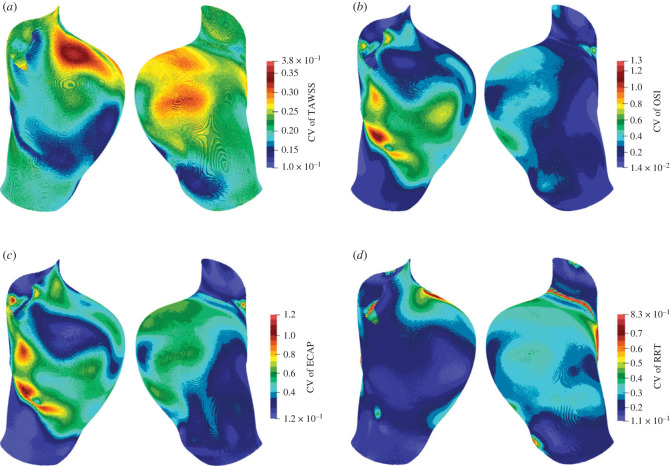
Distribution of the ensemble coefficient of variation (CV) of the risk factors (*a*) TAWSS, (*b*) OSI, (*c*) ECAP and (*d*) RRT on the walls of the abdominal aortic aneurysm. In each panel, the front side of the aneurysm is displayed on the left, and the back side is displayed on the right. The risk factors are averaged over three cardiac cycles in the simulations. These distributions of CV complement the distributions of the mean values in figure 6. For ECAP, the regions with a large mean have a small CV. However, for RRT, some regions with a large mean also have a large CV.

We also calculate the ensemble CV of the risk factors with the aim of assessing the reliability of the above findings. The distributions of CV on the walls of the AAA are shown in figure 7. The CV of TAWSS is about 0.2 in general, although a larger CV is found near the entrance. The distributions of the CV of the OSI and ECAP are very similar. On most surfaces, the CV is below 0.6. Although there are regions with a larger CV, they are regions with lower risk where the mean values are small (figure 6). More importantly, the regions with large mean values correspond to a relatively low CV, suggesting greater confidence that these regions are at a higher risk. However, for RRT, higher values of CV are found in the regions with a large mean value. This implies that the focal regions indicated by RRT may be exaggerated.

In the following analyses, the risk factors have been averaged over space, time, and samples. The CV and CVR of the risk factors are displayed in table 2. The average CVR of 1.87 indicates that our model almost doubles the input uncertainty. The risk factors in increasing order of CVR are OSI, RRT, TAWSS and ECAP. This indicates that the increased sensitivity of ECAP to initial values may offer the greatest flexibility in being able to match observed data.

**Table 2.** Coefficient of variation (CV) and coefficient of variation ratio (CVR) of input parameters and quantities of interest. The CVR of a quantity of interest, given by its CV over the average CV of input parameters, shows the extent to which the uncertainty is amplified. TAWSS, OSI, ECAP and RRT are averaged over the walls of the aneurysm and three cardiac cycles in the simulations.

parameter	CV	parameter	CV	quantity of interest	CV	CVR
Λ	1.086	*m*	0.348	TAWSS	0.971	2.02
Re	0.058	*γ*_*R*_	0.753	OSI	0.673	1.40
Wo	0.057	*γ*_*C*_	0.759	ECAP	1.108	2.30
*E*	0.454	*T*_*s*_	0.576	RRT	0.851	1.77
*F*	0.236					
average:			0.481	average:	0.900	1.87

#### 3.2.3. Sensitivity to input variables

**Figure 8.**
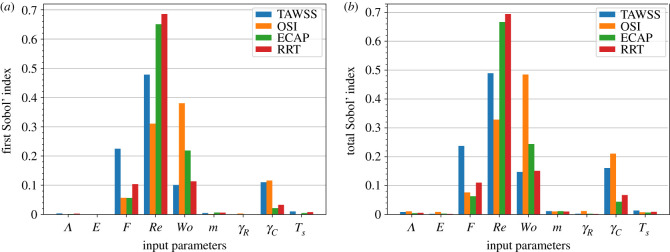
(*a*) First-order and (*b*) total Sobol’ indices of the input parameters with respect to the risk factors for the abdominal aortic aneurysm. The risk factors are averaged over the walls of the aneurysm and three cardiac cycles in the simulations. The first-order indices highlight the need for a more accurate determination of the values of Re and Wo. Considerations could be given to maintaining constant values for Λ, *E*, *m* and *γ*_*R*_ in the model. The results for *T*_*s*_ suggest that the aleatoric uncertainty in the measurements has minor effects on the risk factors.

Next, we examine the relative importance of the input parameters with respect to the risk factors by using Sobol’ indices. In figure 8, the first-order and the total Sobol’ indices are displayed. The differences between the two plots in each column are small, indicating that the input parameters have small interactive effects on the variability of the risk factors. It is clear that the Reynolds and Womersley numbers give the highest values for both indices. Hence, they have a profound influence on model outcomes and should be measured more precisely. Conversely, the parameters *F* and *γ*_*C*_ give lower Sobol’ indices. While they contribute to the variability of the risk factors, their impact pales in comparison to Re and Wo. The other model parameters, Λ, *E*, *m* and *γ*_*R*_, show marginal effects on the variability of the risk factors. Therefore, it is plausible to consider fixing their values in the model. In addition, the results for *T*_*s*_ indicate that the aleatoric uncertainty in the measurements has a minor influence on the risk factors.

**Figure 9.**
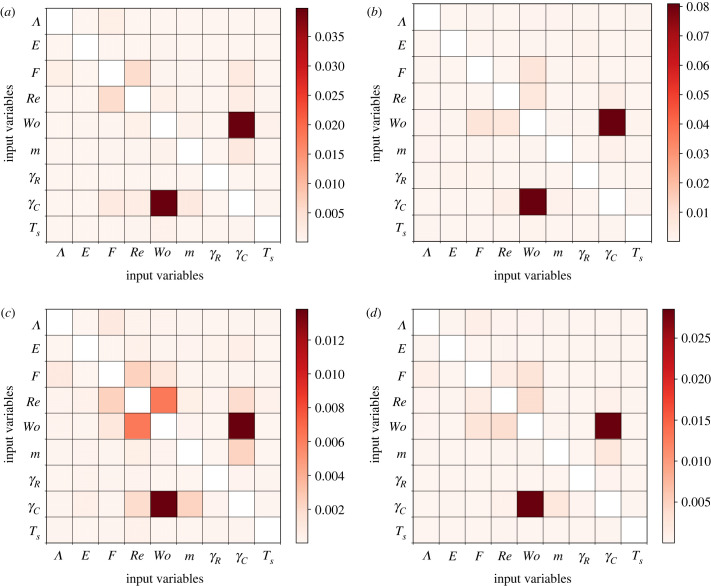
Second-order Sobol’ indices of the input parameters with respect to the risk factors (*a*) TAWSS, (*b*) OSI, (*c*) ECAP and (*d*) RRT for the abdominal aortic aneurysm. The risk factors are averaged over the walls of the aneurysm and three cardiac cycles in the simulations. Note that the plots are symmetric about the diagonal line from the top left to the bottom right. The larger index for Wo and *γ*_*C*_ in all plots indicates that their interactions contribute to most of the second-order variations in the risk factors.

Last but not least, we look into the second-order Sobol’ indices of all pairs of input parameters as shown in figure 9. These indices are small in comparison to the first-order and total Sobol’ indices presented in figure 8. This is consistent with the fact that the differences between the first-order and total Sobol’ indices are small. For all risk factors, the second-order Sobol’ index of Wo and *γ*_*C*_ shows the highest value. Thus, the interaction between these parameters can explain most of the second-order variations in the risk factors. In fact, Wo is related to *γ*_*C*_ via the frequency of the heartbeat profile, as mentioned in §2.2. Secondly, the interactions involving Re are the most prominent; for example, the Sobol’ index of Wo and Re for ECAP ranks second. The other interactions are less important, although they contribute to the uncertainty of the risk factors.

### 3.3. Limitations of current study

As is the case for all simulation studies, assumptions made in the formulation and analysis of our work do place restrictions on the broader applicability of our findings. The first is that this study considers only one specific shape of AAAs. Since vessel walls have significant effects on vascular flows, it is known that different shapes of AAAs induce different flow patterns inside them. Qiu *et al.* [80] identified three types of flow patterns observed in AAAs based on helicity and the number of vortices. These patterns may show different characteristics of the uncertainty in the QoIs and their distribution within AAAs. In addition, if an impinging flow rather than a circulatory flow is developed within the AAA, then the assessment of the risk of growth and rupture will be different. As noted by Meng *et al.* [9], a large magnitude of WSS and a positive WSS gradient are greater risk indicators for impinging flows than for circulatory flows. Analogues for ECAP and RRT, which are defined for circulatory flows, combining these factors should also be calculated to help assess overall risk.

The second limitation is the assumption of a parabolic-shaped spatial profile for the inlet velocity [44]. A few studies have suggested the importance of using a patient-specific three-dimensional velocity profile at the inlet to accurately describe the blood flow in the aorta [81–83]. However, the importance in general becomes less profound as the flow moves downstream due to the accumulated influence of vessel walls. Madhavan & Kemmerling [84] showed that the flow solutions obtained by using inlet profiles of different shapes have small differences beyond two diameters downstream of the inlet. For the arterial model used in this study (figure 1), the inlet is significantly upstream from the region of interest, namely the AAA. Therefore, the difference in the uncertainty of the AAA risk factors by imposing a patient-specific inlet profile is expected to be minor for our studied arterial model. However, further investigation would be required to confirm this hypothesis.

A third limitation is that this study focuses the uncertainty analysis on quantities derived from WSS. There are other hydrodynamic indicators for the risk of rupture of AAAs such as vorticity and helicity, since helical vortices and recirculation zones were found to be associated with ruptures of AAAs [12,80], and wall normal stress, since ruptures can be considered as mechanical failures [1,7]. An examination of a broader range of risk factors would assist in further generalizing our observations. Future campaigns of uncertainty quantification of flows in AAAs could address and investigate the above limitations within the framework of methods presented in this study.

In regard to the modelling of the effects of PAD, we have shown that the outlet boundary condition used can control the flow rates quite accurately. On the other hand, our sensitivity analysis suggests that the flow rates determined from Murray’s power law do not significantly affect the variability of the AAA risk factors. These results imply that Murray’s Law power can be fixed in the simulations of healthy conditions. However, the sample space we studied does not consider the distributions of flow rates in pathological conditions; for example, when there is stenosis, occlusion, or dilation in any particular peripheral artery as for PAD. These conditions are left for future investigations.

Our results reiterate the strength of the link between the fundamental flow parameters Re and Wo, the distribution of flow to peripheral vessels, and the key risk factors associated with the growth and rupture of AAAs. In a clinical setting, this is supplementary evidence towards treatments that seek to keep these within acceptably controlled bounds.

## 4. Conclusion

In summary, this paper introduces a fluid mechanics model designed to simulate the impact of PAD on risk factors associated with AAAs. This model allows us to incorporate abnormal flow rates in peripheral arteries as boundary conditions for blood flow simulations, while the assessment of the risk factors is grounded in quantities of interest derived from WSS.

We address the inherent uncertainty in the risk factors by quantifying it across nine crucial input parameters within a comprehensive arterial model featuring an AAA. The parameters encompass a range of crucial factors, including the characterization of the inlet velocity profile, fluid flow modelling, the influence of elastic wall properties, the regulation of outlet flow rates and the aleatoric uncertainty in measurements for turbulent flow. The uncertainty quantification and the subsequent analysis, conducted on a high-performance computer with the support of an automation toolkit, yield valuable insights.

Our findings demonstrate that flow rates in peripheral arteries can be accurately controlled using two-element Windkessel models, although careful attention is necessary for specific outlets. We reveal that ECAP and RRT provide valuable guidance in identifying regions at higher risk for the growth and rupture of AAAs. Notably, the results of ECAP show greater reliability and adaptability compared to RRT, as evidenced by their coefficients of variation. Additionally, the CVR highlights that uncertainty in these calculations is approximately double that of the input parameters.

Our sensitivity analysis sheds light on the significance of individual parameters. Parameters governing the amplitude and frequency of the inlet velocity exert the strongest influence on the variability of the risk factors and warrant precise determination. Conversely, parameters with limited direct impact on flow characteristics can be held constant.

In conclusion, this study advances the exploration of the haemodynamic interactions between PAD and AAAs using computational fluid dynamics simulations. By quantifying uncertainty and identifying key parameters, this study paves the way for more accurate and efficient simulations, thereby enabling a deeper insight into the underlying dynamics. These insights are pivotal in the development of more effective preventive and treatment strategies, which will ultimately improve outcomes and reduce mortality rates for individuals affected by PAD and AAAs.

## Data Availability

Supplementary materials including the supplementary text, the arterial model, the code used to perform the campaigns of uncertainty quantification and all the results of the campaigns is available from the Figshare repository: https://doi.org/10.5522/04/c.6871825 [85].
